# Exploitation of the chick embryo chorioallantoic membrane (CAM) as a platform for anti-metastatic drug testing

**DOI:** 10.1038/s41598-020-73632-w

**Published:** 2020-10-09

**Authors:** P. Pawlikowska, T. Tayoun, M. Oulhen, V. Faugeroux, V. Rouffiac, A. Aberlenc, A. L. Pommier, A. Honore, V. Marty, O. Bawa, L. Lacroix, J. Y. Scoazec, A. Chauchereau, C. Laplace-Builhe, F. Farace

**Affiliations:** 1 U981 “Identification of Molecular Predictors and New Targets for Cancer Treatment”, INSERM, 94805 Villejuif, France; 2 “Circulating Tumor Cells” Translational Platform, UMS AMMICa CNRS 3655-INSERM US23, Gustave Roussy, Université Paris-Saclay, 94805 Villejuif, France; 3 Flow Cytometry and Imaging Platform, UMS AMMICa CNRS 3655-INSERM US23, Gustave Roussy, Université Paris-Saclay, 94805 Villejuif, France; 4 Genomic Platform, CNRS UMS3655 – INSERM US23 AMMICA, Gustave Roussy, Université Paris-Saclay, 94805 Villejuif, France; 5 Experimental and Translational Pathology Platform, CNRS UMS3655 – INSERM US23 AMMICA, Gustave Roussy, Université Paris-Saclay, 94805 Villejuif, France

**Keywords:** Cancer models, Metastasis

## Abstract

The establishment of clinically relevant models for tumor metastasis and drug testing is a major challenge in cancer research. Here we report a physiologically relevant assay enabling quantitative analysis of metastatic capacity of tumor cells following implantation into the chorioallantoic membrane (CAM). Engraftment of as few as 10^3^ non-small cell lung cancer (NSCLC) and prostate cancer (PCa) cell lines was sufficient for both primary tumor and metastasis formation. Standard 2D-imaging as well as 3D optical tomography imaging were used for the detection of fluorescent metastatic foci in the chick embryo. H2228- and H1975-initiated metastases were confirmed by genomic analysis. We quantified the inhibitory effect of docetaxel on LNCaP, and that of cisplatin on A549- and H1299-initiated metastatic growths. The CAM assay also mimicked the sensitivity of *ALK*-rearranged H2228 and *EGFR-*mutated H1975 NSCLC cells to tyrosine kinase inhibitors crizotinib and gefitinib respectively, as well as sensitivity of LNCaP cells to androgen-dependent enzalutamide therapy. The assay was suggested to reconstitute the bone metastatic tropism of PCa cells. We show that the CAM chick embryo model may be a powerful preclinical platform for testing and targeting of the metastatic capacity of cancer cells.

## Introduction

Metastasis is the major cause of death from cancer. Uncovering new therapeutic targets in metastatic cancer inevitably relies on suitable and functional models amenable to pharmacological assays. Different immunodeficient mouse strains allowed human cells engraftment and opened possibilities for developing models. Over the past few years, tremendous effort has been put into establishing different patient-derived xenografts (PDX) to model patient disease and decipher novel therapeutic strategies^1^. PDX are currently the most clinically relevant models but difficulty to obtain the metastatic growth from PDX tumors makes functional studies and screening approaches challenging. Depending on research purposes, diverse sites of tumor engraftment were applied, such as orthotopically or directly in the vascular system. However, both rarely reflect the real patient metastatic disease^1^. Furthermore, costs related to establishment and husbandry of mouse models and ethical issues considerably limit their use. Recently, 3D organoids have emerged as novel robust tools to model tumor heterogeneity and perform drug screening assays^2^. Great efforts have been put into generating 3D co-cultures to simulate tumor microenvironment ex vivo. However, the lack of in vivo host complexity renders modeling metastatic disease incomplete. To overcome some of these limitations and to bridge the gap between in vitro and in vivo study of metastasis, we and others attempted to use a chorioallantoic membrane (CAM) chick embryo model.

The first applications of the chick embryo and CAM in oncology research were announced more than a century ago^3,4^. The chicken egg model has fundamentally contributed to the most significant discoveries and some Nobel laureates, including the discovery of the first known oncogene (c-src)^5^. The chick embryo develops for 21 days until hatching. The CAM is formed within 4 to 5 incubation days (ID) through the fusion of mesodermal layers of outgrowing allantois and the chorion. The highly vascularized nature of the CAM is a considerable advantage, it greatly stimulates the growth of grafted cells. Notably, this avian model is a naturally immunodeficient host; a feature which allows implantation of tumor cells and tissues without species-specific restrictions. The extraembryonic membranes connected to the embryo through a continuous extraembryonic vessel system are easily accessible for manipulation and observation. According to European law (Directive 2010/63/EU of the European Parliament and of the Council of 22 September 2010 on the protection of animals used for scientific purposes), the CAM model system does not raise any ethical or legal concerns, thus being an attractive alternative to other animal experiments. The CAM model is maintained in an incubator at 37 °C requiring limited space. This significantly limits animal husbandry requirements compared to immunodeficient mice breeding and reduces experimentation costs. The availability of the CAM model for screening is beneficial compared to in vitro cultures, where the role of tumor vasculature and tissue tropism cannot be accounted for.

Several previous reports show the feasibility of obtaining tumor growth in the form of nodules on the CAM, starting from different tumor cell lines or tissues^4,6,7^. These studies usually aim to evaluate the morphological and morphometric characteristics of tumor nodules. Indeed, tumor growth has been evaluated by measuring the size and weight of nodules as well as the extent of the vascular network^8,9^ or by immunohistochemical studies on isolated CAM primary tumors^10,11^. Technological development, especially in the field of imaging, opened up new possibilities to quantify the primary tumor growth^11,12^ and study tumor cell migration^13,14^. Up until now, metastatic tumor evolution was analyzed by quantitative Alu-PCR for the detection of human tumor cells in the isolated organs of the chick embryo^15,16^. The fluorescent tumor cells were also detected at tissue sections of isolated organs^17,18^. Here, we investigated whether the CAM model may be used to evaluate the metastatic capacity of cells and serve as a potential preclinical anti-metastatic drug test. We report a step by step technology to obtain metastatic growth starting from a limited number of well-characterized tumor cells. We show the quantitative assessment of metastasis using whole-animal 3D tomography optical imaging. Finally, we provide a proof-of-concept for the feasibility of a preclinical assay of anti-metastatic compounds in the chick embryo model.

## Results

### Primary tumor nodule formation starting with limited cell quantities

**Figure 1:**
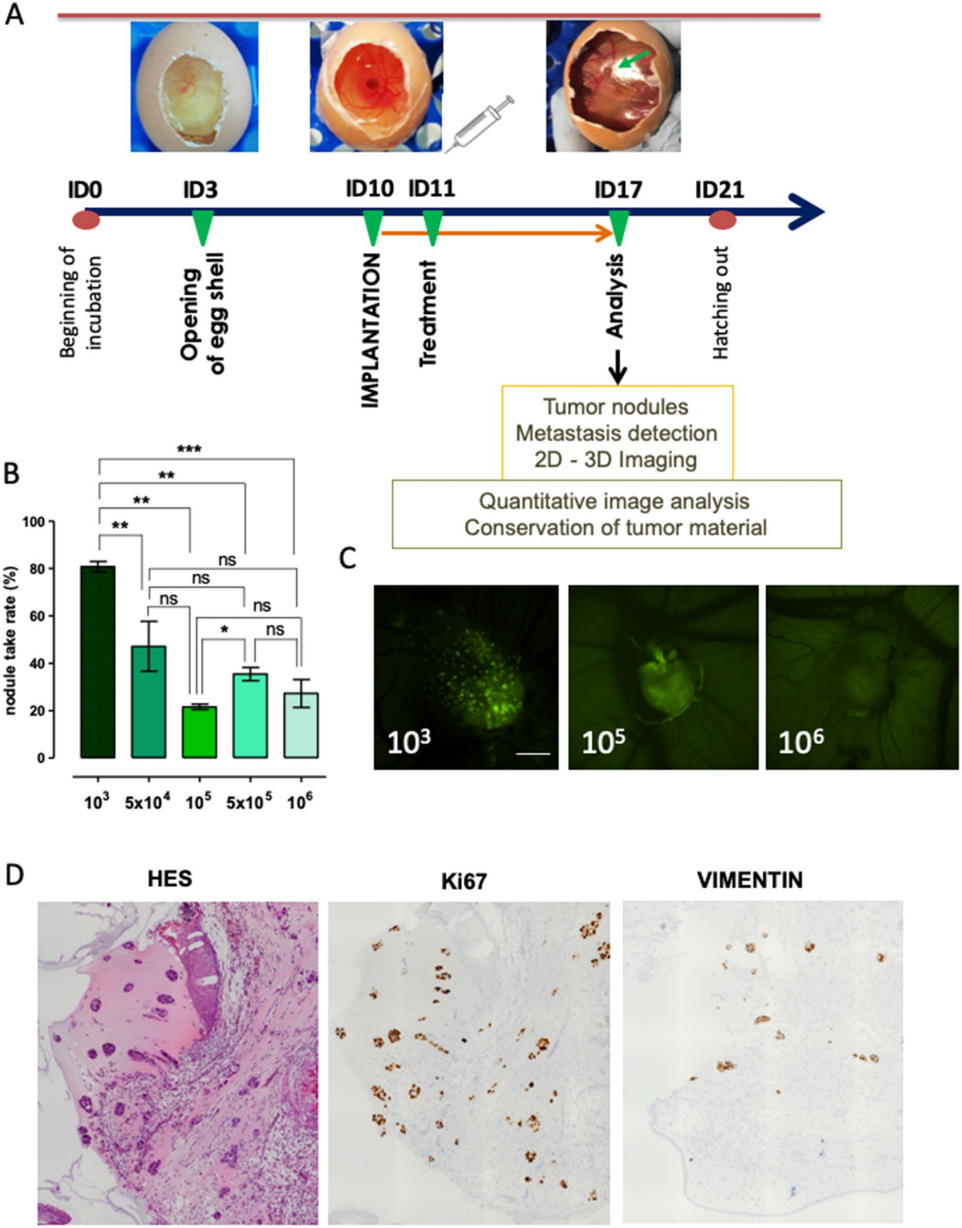
Chorioallantoic membrane (CAM) chick embryo model and primary tumor detection. (**A**) Schematic model embedding our timeline of standard experiment, supplemented by the images representing incubation day (ID) 3, 10 and 17. (**B**) Graph displaying percentage of eggs presenting fluorescence-positive nodules at ID 17 after implantation of indicated number of cells. Data are represented as mean ± SEM obtained from 14 experiments with 10^3^ cells, 4 experiments with 5 × 10^4^, 10^5^ and 5 × 10^5^ cells, and 8 experiments with 10^6^ cells. In each experiment, a minimum of 8 eggs were evaluated. (**C**) Representative images of the fluorescent tumor nodules of H1299-GFP obtained at ID 17. (**D**) Tumor nodules obtained at ID 17 after implantation of 10^3^ H1299-GFP cells, FFPE sections stained with HES, anti-human Ki67 and anti-human Vimentin antibodies.

Most previous studies showed the advantages of cell implantation at ID 10 for primary tumor nodule formation^4,6,8^. We adapted a similar protocol to a panel of non-small cell lung cancer (NSCLC) and prostate cancer (PCa) cell lines (Supplementary Table 1). The general scheme followed in all experiments is presented in Fig. 1A. In contrast to previous research, we challenged the eggs with a highly limited number of H1299 cells starting from 10^6^ and successively reducing cell quantities (Fig. 1B). Reproducible results were obtained by implantation of 10^3^ cells per egg, resulting in up to 80% of successfully formed nodules in the case of lung cancer cell lines (Fig. 1B) and 100% for prostate cancer cell lines (data not shown). Engrafted cell lines were engineered to express fluorescent markers. Tumor growth was evaluated by fluorescence detection in vivo using MacroFluo Nikon macroscope (Fig. 1C). Hematoxylin staining together with anti-human antibodies against Ki67 and tumor marker vimentin was further performed to confirm the human origin of tumor growth on the CAM (Fig. 1D). These results demonstrate the ability of the CAM system to form tumor nodules from as low as 10^3^ cells.

### Evaluation of metastasis formation in the chick embryo

**Figure 2:**
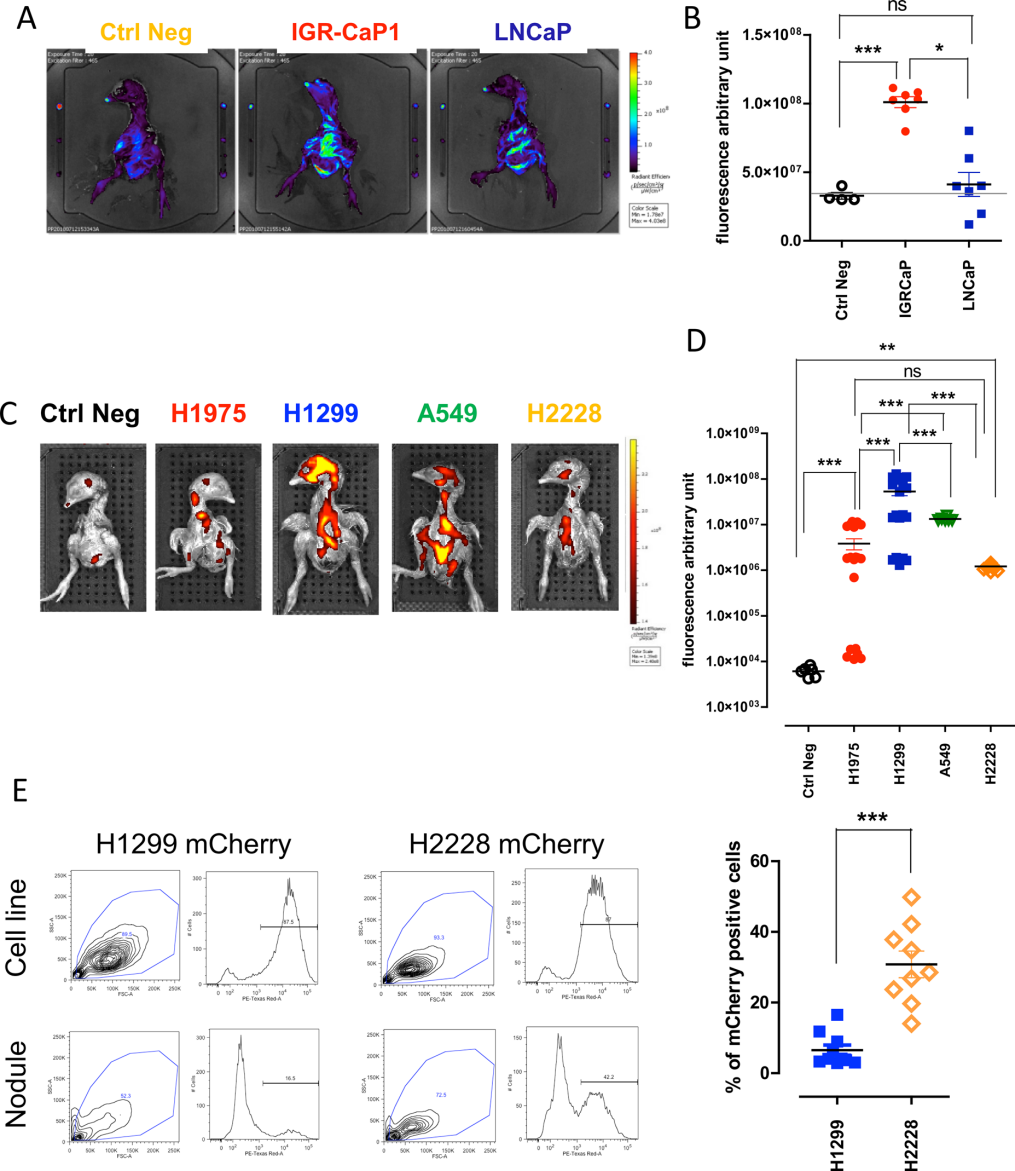
Metastatic capacities of prostate and lung cancer cell lines. (**A**) 2D representative images of chick embryo expressing GFP originating from eggs implanted at ID 10 with medium only (without cells, left), IGR-CaP1-GFP (middle), LNCaP-GFP (right). (**B**) Quantitative analysis of average fluorescence intensity of chick embryos presenting PCa metastases measured after 2D scan. Each point represents a single embryo. Two separate experiments were performed. (**C**) 2D representative images of chick embryos expressing mCherry originating from eggs implanted at ID 10 with NSCLC cell lines. (**D**) Quantitative analysis of average fluorescence intensity obtained after 3D scans of chick embryos presenting metastases formed after implantation of lung cancer cell lines. At least two separate experiments were performed for each cell line. Each point represents a single embryo. Numbers of analyzed embryos were for Negative Controls: 6, H1299: 21, H1975: 8, A549: 6, H2228: 14. (**E**) Representative FACS plots obtained after mCherry expression analysis in control in vitro cell lines and tumor nodules obtained at ID 17 from CAM implanted with H1299-mCherry and H2228-mCherry respectively. Right panel represents graphical quantification of H1299 and H2228 mCherry-expressing cells obtained from nodules at ID 17 after mechanistic and enzymatic dissociations. Two separate experiments were performed for each cell line. Each point represents a single embryo.

We then focused on the quantitative evaluation of metastatic seeding capacities of tumor cells. As the duration of the CAM assay is limited to 7–9 days before the chick hatches, most tumor cells cannot produce macroscopically visible tumor metastasis masses before the end of the assay. However, they form small metastatic foci detectable by fluorescence imaging. We thus aimed to adapt the imaging tomography IVIS Spectrum system—most often used for visualizing tumor growth in small rodents—to detect the metastases formed by fluorescent cell lines implanted on the chick embryo. We first engrafted the GFP-expressing LNCaP and IGR-CaP1 PCa cells (10^3^ cells), for which the tumorigenic potential has been reported in mice^19–21^. LNCaP primary nodules were also reported to grow on the CAM^9^. As previously observed in mouse models^19,20^, IGR-CaP1-GFP appeared to display significantly increased disseminating capacity compared to standard androgen-dependent LNCaP-GFP cell line, as measured by fluorescence intensity obtained using 2D scans performed by IVIS Spectrum system (Fig. 2A,B). However, significant autofluorescence of the chick embryo was repeatedly observed throughout the analysis of GFP-expressing cells, which can hamper signal interpretation for less aggressive disseminating cells when using the GFP spectral range. Indeed, some of the embryos that were implanted with LNCaP-GFP cells presented fluorescence intensity equal to the one in negative control embryos, making the interpretation difficult. Since green fluorochrome is often used in in vivo research, we would like to point out that based on these results, GFP expression—although well-distinguished in the tumor nodule on the CAM—is not really suitable to evaluate the metastasis seeding inside the chick embryos using the fluorescence scan system. As much more limited autofluorescence is obtained in the red fluorescence spectra, further experiments were pursued with mCherry-expressing cells (after retroviral infection, Fig. 2C) or PKH26-stained cells (described below, Supplementary Table 2, Supplementary Figures 2 and 3). The protocol of cell labeling with fluorescent cell linker PKH26 was adapted to extend the use of the model for tumor cells that were not engineered to express fluorescent markers. Engraftment of 10^3^ mCherry-expressing NSCLC cells produced identifiable metastatic foci (Fig. 2C,D). The most aggressive cell line was *NRAS*-mutated H1299. *KRAS*-mutated A549, *EGFR*-mutated H1975 and *ALK*-rearranged H2228 cell lines also produced significantly higher fluorescent signals compared to negative control chick embryos (Fig. 2C,D). Notably, heterogeneity in the mean fluorescence of metastatic foci from H1975 cells was observed. Average fluorescence intensity was measured in a region of interest (ROI) including the whole embryo. To further confirm metastasis foci formation, we investigated known H1975 and H1299 mutations (Supplementary Table 1) in CAM nodules and chick embryo metastases, in comparison to control cell lines. *EGFR*^*T790M*^, *EGFR*^*L858R*^ and *TP53*^*R273H*^ homozygous mutations were detected in tested tumor nodules and metastasis samples from chick embryos engrafted with H1975 cells (Table 1). Furthermore, we detected homozygous *CDKN2A*^E69*^mutation in H1975 metastatic samples. In the case of H1299, the most aggressive cell line according to fluorescence measurements in the chick embryo, we detected the characteristic heterozygous mutation *NRAS*^*Q61K*^ only in the organs invaded by metastasis and not in primary nodules (Table 1). This suggested that, at ID 17, all H1299 cells from the primary tumor site had migrated to embryo organs. To further evaluate whether some cell lines presented higher migration capacities than others, we implanted, in parallel, H1299-mCherry cells—the most aggressive cells according to fluorescence analysis of the chick embryo—and the less aggressive H2228-mCherry cells. Indeed, the metastatic fluorescence signal obtained from the chick embryos at ID 17 was statistically different (Fig. 2D). Moreover, dissociated primary nodules evaluated by FACS from the same eggs presented negative correlation with the fluorescent metastatic foci intensities. Less mCherry-positive cells were found in primary nodules of aggressive H1299 cells compared to H2228, which were predominantly present in nodules but displayed lower intensity of fluorescent metastatic foci in corresponding chick embryos (Fig. 2E).

**Figure 3:**
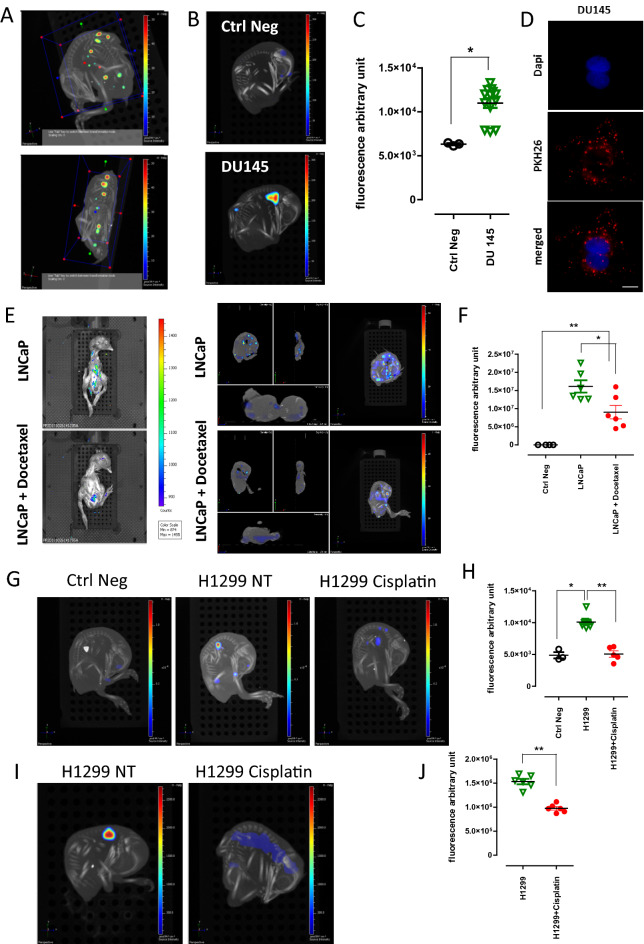
Effect of chemotherapeutics on metastasis formation. (**A**) Representation of the methodology adapted to fluorescence intensity measurement on 3D reconstructed images. Region of interest was defined manually, using an adapted embryo size area in each case (blue box). (**B**) 3D representative images of chick embryos expressing PKH26 (bottom) 7 days after implantation with DU145 PKH26-positive cells. (**C**) Quantitative analysis of PKH26 positive and negative chick embryos. Each point represents a single embryo. (**D**) Representative images of DU145 cells 7 days after induction with PKH26 fluorescent tracer. Bar = 10 µm. (**E**) Representative images of 2D (left) and 3D (right) reconstructions of chick embryos obtained from eggs implanted with LNCaP-mCherry cells with or without docetaxel treatment. (**F**) Quantitative analysis of mean fluorescence intensity on 3D reconstructed images. Each point represents a single embryo. (**G**) Representative images of fluorescent metastatic foci seeded by H1299 cells with or without cisplatin treatment. (**H**) Quantitative analysis of mCherry-positive embryos obtained from implantation of H1299 mCherry-expressing cells, with or without cisplatin treatment. At least two separate experiments were performed for each therapeutic treatment and the graph presents the most representative one. (**I**) Representative images of metastatic foci obtained after injection of H1299-mCherry cells directly into the chick embryo circulation and treatment with or without cisplatin. (**J**) Quantitative analysis of mean fluorescence of chick embryos obtained from two separate experiments after injection of H1299-mCherry cells directly into the chick embryo circulation and treatment with or without cisplatin.

**Table 1:** Genomic analysis of tumor nodules and metastases as well as corresponding cell lines with NGS Ampli1 CHP Custom (Menarini Silicon Biosystems) and home-made NSCLC Panel.

Cell line	Sample	Gene	Protein change	Exon	Variant Allele frequency (%)
NCI-H1975	gDNA	*EGFR*	T790M	20	76
		*EGFR*	L858R	21	75
		*TP53*	R273H	8	100
		*CDKN2A*	E69*	2	100
	Nodule N1	*EGFR*	T790M	20	52
		*EGFR*	L858R	21	67
		*TP53*	R273H	8	100
		*CDKN2A*	E69*	2	Not covered
	Nodule N4	*EGFR*	T790M	20	19
		*EGFR*	L858R	21	32
		*TP53*	R273H	8	75
		*CDKN2A*	E69*	2	Not covered
	Metastasis M1	*EGFR*	T790M	20	79
		*EGFR*	L858R	21	78
		*TP53*	R273H	8	99
		*CDKN2A*	E69*	2	100
	Metastasis M2	*EGFR*	T790M	20	76
		*EGFR*	L858R	21	75
		*TP53*	R273H	8	100
		*CDKN2A*	E69*	2	100
	Metastasis M3	*EGFR*	T790M	20	74
		*EGFR*	L858R	21	74
		*TP53*	R273H	8	100
		*CDKN2A*	E69*	2	100
	Metastasis M4	*EGFR*	T790M	20	73
		*EGFR*	L858R	21	72
		*TP53*	R273H	8	100
		*CDKN2A*	E69*	2	100
NCI-H1299	gDNA	*NRAS*	Q61K	3	44
	Nodule N1	*NRAS*	Q61K	3	0
	Nodule N3	*NRAS*	Q61K	3	0
	Metastasis M1	*NRAS*	Q61K	3	35
	Metastasis M2	*NRAS*	Q61K	3	40
	Metastasis M3	*NRAS*	Q61K	3	45
	Metastasis M5	*NRAS*	Q61K	3	44

Fluorescent imaging acquisitions providing 3-dimensional (3D) reconstitution of the embryo allowed us to precisely evaluate metastasis formation both in terms of localization and quantification (Fig. 3A). The acquisition of 3D computer tomography (CT) data combined to fluorescence images aids to localize the metastatic foci in the chick embryo and thus determine the organ with tumor lesions (Fig. 3A). Fluorescent signal was collected in specified wavelengths during serial acquisitions to filter specifically the fluorescence from GFP-, mCherry- or PKH26-positive cells. Here, we provided the example of PCa cell line (DU145) labeled with PKH26 and either cultured in vitro or implanted into the CAM (2 × 10^3^ cells per egg). We detected the fluorescent signal from metastatic foci of the chick embryo (Fig. 3B,C; Supplementary Figure 3C) and cultured cells (Fig. 3D, Supplementary Figure 2) 7 days later. These results show that 3D reconstitution of chick embryos allows us to detect even a limited number of fluorescent cells in the whole embryo, suggesting that our system might be used as a preclinical model of metastasis.

### Effect of the chemotherapeutics on metastasis formation in the chick embryo

Since we have shown the feasibility of using chick embryo as an efficient and sensitive model for metastasis seeding for different type of cancers, we thought that it might be particularly useful to assess therapeutics targeting metastasis. We implanted LNCaP-mCherry cells into the CAM at ID 10. Docetaxel (2 µg/kg), standard-of-care for PCa patients, was topically added on the surface of the CAM 24 h later (ID 11). The eggs were analyzed 6 days later (ID 17). The dose of docetaxel has been previously estimated by serial treatments (data not shown). Both 2D and 3D imaging showed that cell migration and metastasis formation into distant organs of the chick embryo was hampered by docetaxel treatment (Fig. 3E). We quantified the effect of docetaxel on the metastasis seeding capacities of LNCaP by measuring the average fluorescence intensity from images obtained after 2D scanning of chick embryos (Fig. 3F). These data showed a significant decrease in fluorescence intensity between chick embryos engrafted with LNCaP versus LNCaP treated by docetaxel.

A similar experiment was performed with NSCLC A549 and H1299 cell lines which are known to be sensitive to cisplatin treatment *in vitro*^22^. Implantation of 10^3^ A549 or H1299 mCherry-expressing cells resulted in the emission of fluorescence signals on 3D reconstructed images 7 days later in different organs of the chick embryo (Fig. 3G, Supplementary Figure 1B). Treatment of chick embryos with cisplatin (10 µg/kg), 24 h after cell implantation, significantly decreased the fluorescence signal from both tested NSCLC cell lines (Fig. 3H, Supplementary Figure 1C). Cisplatin dose did not affect neither viability nor development of embryos. For all evaluated drugs chick embryo mobility as well as increasing vascularization of the CAM (the two indicators of chick embryo viability) were evaluated and compared to non-treated eggs throughout experiments. The effect of the therapeutics on chick embryo development was also estimated. Distinctly smaller embryonic size, unilateral or bilateral microphthalmia, microcephaly or ancephaly, acrania, malformations of limbs, gastroschisis or other visible malformations if detected in more than 10% of treated embryos, the drug dose was decreased.

To demonstrate that the observed reduction of metastatic signal after cisplatin treatment is not simply the result of tumor cell death at the primary site, we directly injected tumor cells into the chick embryo circulation to bypass the stage of primary tumor formation. This technically-advanced manipulation evidently increases the mortality of chick embryos. However, protection of the manipulated vein by silicon ring facilitated the healing process and allowed an adequate chick embryo development, as well as metastatic growth. No primary nodules were observed on the CAM in this experiment, however fluorescent metastatic foci were found in the chick embryo injected with mCherry-expressing H1299 cells (2 × 10^3^) and fluorescence of less intensity was detected after cisplatin treatment (Fig. 3I,J). These data showed that the CAM chick embryo system is a sensitive model capable of recapitulating the effect of standard-of-care chemotherapies frequently used against metastatic PCa and NSCLC.

### Effects of targeted therapies on metastasis formation

**Figure 4:**
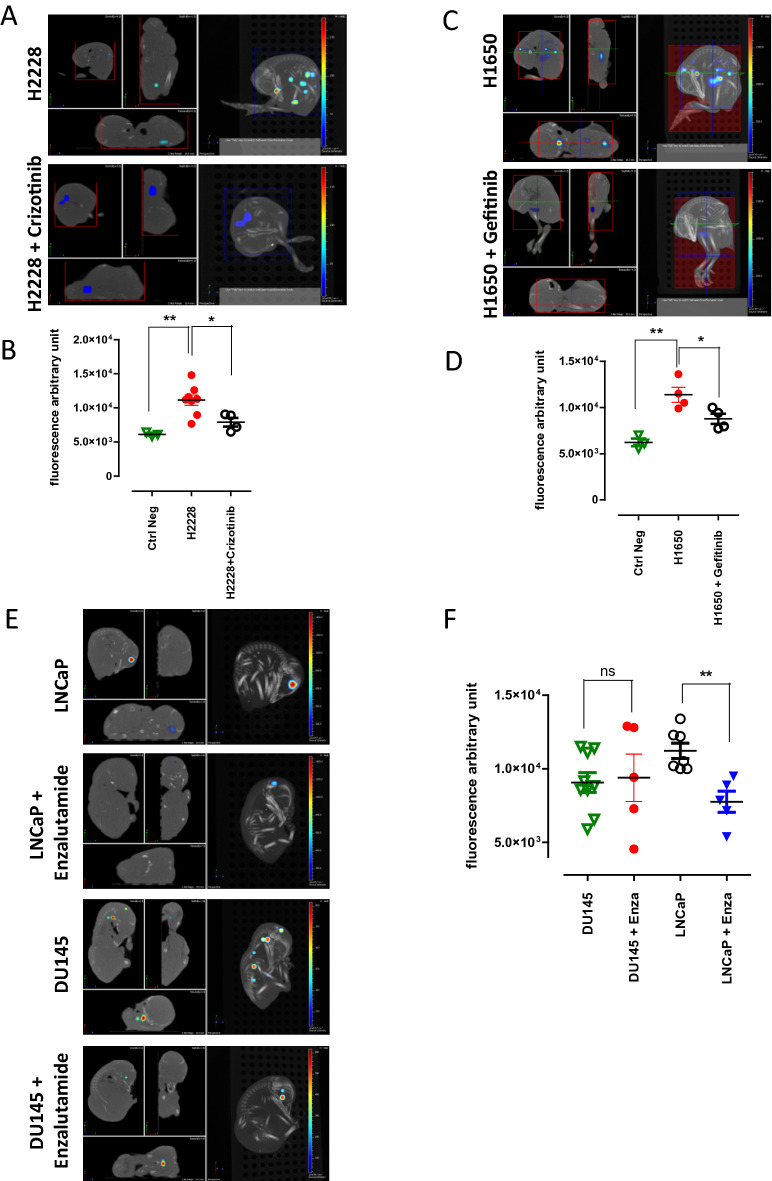
Effect of targeted therapies on metastasis formation. (**A**) Representative images of 3D views of chick embryos originating from eggs implanted with H2228 mCherry-expressing cells with or without crizotinib treatment. (**B**) Analysis of chick embryos coming from eggs implanted with H2228-mCherry with or without crizotinib. (**C**) Representative images of 3D views of chick embryos from eggs implanted with H1650 cells with or without gefitinib treatment. (**D**) Analysis of metastasis from eggs implanted with H1650 cells with or without gefitinib treatment. At least two separate experiments were performed for each therapeutic treatment and the graph presents the most representative one. (**E**) 3D representative images of chick embryos expressing PKH26 and originating from eggs implanted at ID 10 with LNCaP or DU145 PKH26-positive cells; with or without enzalutamide treatment. (**F**) Quantitative analysis of average fluorescence intensity from eggs implanted with PCa cells, with or without enzalutamide treatment. Each point represents a single embryo. Graph represents results obtained from two separate experiments.

Then we wondered if targeted therapy efficacy could also be evaluated using the CAM system. We established first a safe dose of 2 mg/kg for tyrosine kinase inhibitors crizotinib and gefitinib (data not shown). As previously, 10^3^ mCherry *ALK*-rearranged H2228 and *EGFR*-mutant H1650 NSCLC cells were implanted into the CAM at ID 10. Similarly to patients’ therapy, groups of eggs were treated daily with respective therapeutics. 7 days later, the embryos treated with targeted therapies presented a significantly lower fluorescence intensity compared to non-treated ones (Fig. 4A-D), showing the inhibitory effects of crizotinib and gefitinib on the metastatic growth of H2228 and H1650 cell lines respectively.

Finally, the metastatic capacity of androgen-responsive LNCaP and—unresponsive DU145 PCa cells was challenged with enzalutamide (400 µg/kg). Before implantation, cells were stained with PKH26 fluorescent tracer. The pattern and intensity of PKH26 labeling in LNCaP cell line were analyzed in vitro for 7 days, the length of chick embryo experiments (Supplementary Figure 2) and no substantial differences were observed. The characteristic punctate pattern of staining was maintained throughout the whole experiment. 2 × 10^3^ cells were implanted per egg into the CAM at ID 10. Enzalutamide was topically administered daily for 6 days starting 24 h after cell implantation. LNCaP and DU145 both formed metastasis in the chick embryo bodies at ID 17 (Fig. 4E,F). As expected, the androgen-unresponsive DU145 cells remained resistant to enzalutamide treatment, while the drug statistically diminished metastatic capacities of LNCaP cells (Fig. 4E,F). Overall, these results demonstrate that the CAM system might be successfully used for efficacy evaluation of targeted therapies in NSCLC and PCa cells.

### The chick embryo represents the specificity of PCa metastatic bone tropism

**Figure 5:**
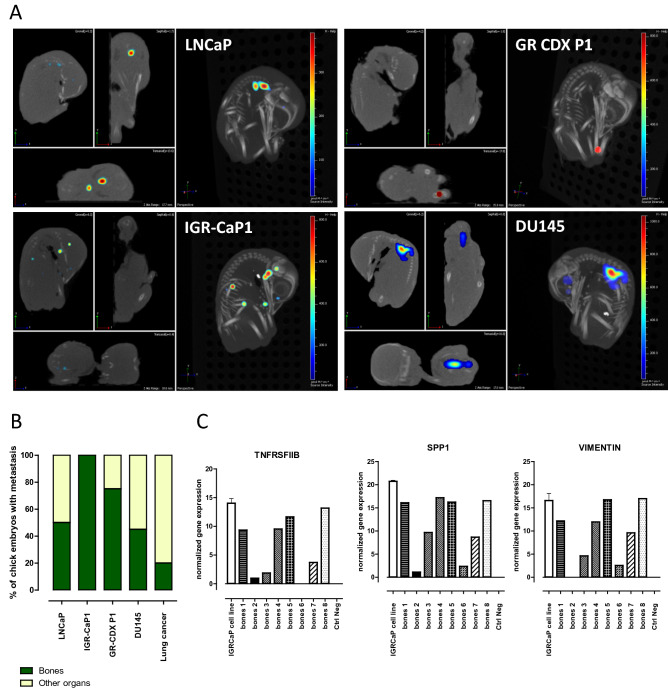
Prostate cancer tropism in chick embryo. (**A**) Representative examples of 3D reconstructions of chick embryos presenting bone-localized metastatic foci. (**B**) Proportions of metastatic sites recovered in bones of the chick embryo. Number of evaluated embryos for LNCaP: 10; IGR-CaP1: 13, GR-CDX P1: 8; DU145: 11; lung cancer: 28. The analysis was performed retrospectively from the images collected during different experiments using PCa cells. (**C**) qRT-PCR analysis of bones isolated from chick embryos after implantation of IGR-CaP1-GFP cells, expression of human TNFRSFIIB, SPP1 and Vimentin was normalized to negative control of bones from non-implanted chick embryos. The positive control was an in vitro collected IGR-CaP1 cell line. Experiment was performed twice with two different negative and positive controls and 4 bones tested in each.

Difficulty to obtain a representative model of PCa bone metastasis hampers the development of new treatments for metastatic PCa patients. To evaluate whether the chick embryo may represent a relevant model of PCa metastatic evolution, we retrospectively compared the 3D reconstitution images of metastatic foci seeded by LNCaP, IGR-CaP1, DU145 cells to metastases formed by the NSCLC cell lines (H1975, H1299, A549, H2228 and H1650). Additionally, we tested GR-CDX P1, a unique cell line established in the laboratory from circulating tumor cell-derived explant (CDX) model of castration resistant prostate cancer (CRPC)^23^. LNCaP cells were found to metastasize to the bones of the chick embryo with 50% incidence (Fig. 5A,B). The more aggressive IGR-CaP1 cells were always localized within the bones. 75% and 54% of tumor foci obtained after implantation of GR-CDX P1 and DU145 cells respectively were found co-localized with the skeleton after image reconstruction. Indeed, this evaluation is rather approximative and based on the overlapping fluorescent spot with the CT image of the chick skeleton. However, the same type of evaluation using lung cancer cell lines shows that metastatic foci disseminated in the bones never exceed 20%, for all cell lines tested. To further confirm the presence of human PCa metastatic foci in the bones of the chick embryo, we collected the long bones from chick embryos implanted with IGR-CaP1 cell line. Bones were cleaned and RNA was extracted from the flushed bone marrows and crashed bones. We detected the expression of human genes that were described as characteristics of the metastatic process of this cell line^19^. 6 bone samples out of 8 presented high expression of TNFSRFIIB, SPP1 and Vimentin evidencing the presence of human tumor cells inside the chick embryo bones. Samples marked ‘bones 2′ and ‘bones 6′ most probably contained very few IGR-CaP1 cells since only two genes out of three tested were found at detectable levels (Fig. 5C). Although requiring further investigation, these data suggest that the CAM chick model may potentially represent a system able to reconstitute the particular metastatic tropism of prostate tumors to the bones.

## Discussion

Further development in the establishment of novel in vivo preclinical models is needed to increase knowledge about tumor seeding and offer new insights into therapeutic strategies. In this study we show that metastases in the organs of the chick embryos can be obtained following the engraftment of a number as low as 10^3^ of tumor cells into the CAM. Standard 2D and 3D optical tomography imaging enabled highly sensitive detection of fluorescent metastatic foci within 7 days from cell engraftment. Metastasis formation in the chick embryo was confirmed by molecular studies. We quantified the inhibitory effects of a variety of standard-of-care treatments including chemotherapy and targeted therapies using several NSCLC and PCa cell lines. We also observed that the CAM chick embryo system may allow the reconstitution of the bone metastatic tropism of PCa cell lines. Our data show that the CAM system may provide a rapid and sensitive method to evaluate the metastatic activity of cancer cells and assess the efficacy of anti-metastatic compounds.

Until now, analysis of tumor growth on the CAM has relied on visual inspection by microscopy and quantification of nodule size or weight^8,9^. These methods have been continuously optimized. Two studies have reported the successful measurement of bioluminescence of human tumors growing in the CAM^11,12^. The dynamics of primary tumor growth using a luciferase reporter was shown for PCa and osteosarcoma^12^. In our work, the primary nodule growth was visualized, similarly to others^10,17,24^, by fluorescence. Microscopic evaluation of primary tumors on the CAM reveals extremely dynamic cellular interactions between fluorescent tumor cells and the microenvironment^13^. During the first days of incubation, the CAM contains three layers: the ectoderm attached to the shell membrane, the mesoderm which is enriched in blood vessels and the endoderm. At ID 10, when tumor cells are implanted, the ectoderm of the CAM is characterized by a highly developed capillary system, which is crucial for tumor seeding and dissemination. An improved technique of microscopic assessment of tumor angiogenesis confirmed these observations using CAM optical sectioning next to classic histological and fluorescent stainings^25^. Tumor cell motility and migration were visualized in the CAM using intravital imaging^10,13^. This methodology was particularly useful for studying the initial migration of cells on the CAM and required large numbers of implanted cells varying from 10^6^^25^ up to 8 × 10^[610^. The lower cell number implanted into the CAM to follow metastatic foci formation started at 2 × 10^4^ per egg^9^. However the most frequently used concentration of engrafted tumor cells was 2 × 10^6^ per egg^18,26^. Here we described a protocol which requires only 10^3^ tumor cells to obtain visible nodule growth on CAM and metastasis development in the chick embryo. This result highlights the sensitivity of our method and detection technique, which may have a particular importance in applications with limited number of cells. For example, this approach may be of interest to evaluate rare subpopulations of cancer stem cells or genetically modified tumorigenic subclones.

Our protocol is both highly favorable for metastasis establishment and sensitive for their detection compared to other studies using the CAM system. We adapted the number of tumor cells able to survive the period of implantation and healing using the growth factor-reduced matrigel medium. Importantly, we applied a micro-hemorrhage induction step to facilitate tumor cell migration. This procedure increases the risk of chick embryo mortality but is nonetheless crucial for stimulation of vascularization, tumor cell migration and seeding. Additionally, we noticed that a high number of rapidly proliferating cancer cells were accompanied by necrosis in the primary nodule. Evidently, the capacity to form metastatic foci is highly dependent on cell type as we showed in the case of two different lung tumor cell lines (H1299 and H2228). Although implantation conditions were exactly the same, the two cell lines differed in metastatic seeding capacities. Finally, the sensitivity of fluorescence detection of the imaging technique used is also an important factor.

To increase our knowledge on metastatic seeding, a deeper observation into the chick embryo is required. However, due to the presence of eggshell, most visualization techniques are usually limited to the CAM and its closely attached areas. Previously reported metastasis formation in the chick embryo was evaluated by immunohistochemical analysis of organ sections^10,27–29^. Otherwise, the tumor burden was estimated by qPCR of ALU sequences from DNA of isolated organs^15,16^. Today, the chick embryo and metastatic cells may be visualized thanks to sensitive imaging modality. The first application of computer tomography (CT) was reported in 2013. CT imaging was combined with PET in order to visualize the tumor tracer uptake^30^. Another interesting report showed that tumor cells labeled with magnetic polymers might be visualized in different organs of the chick embryo using Magnetic Resonance Imaging (MRI^31^). However, this method requires an adapted contrast agent and a high number of cells to be implanted on the CAM, due to the substantial loss of signal resulting from intensive cell proliferation. In some cases, to avoid the eggshell barrier, experiments were performed *ex ovo* to present dynamic tumor cells motility by videomicroscopy^14^. In the *in ovo* experiments reported here, since fluorescence signals are undetectable throughout the eggshell, we measured metastatic foci *post mortem* inside the chick embryo using 3D fluorescence imaging. Additionally, combining fluorescence acquisition to 3D CT allowed the specific localization of metastatic foci in the embryo. It is important to state that, as observed using most *in ovo* techniques, our method does not directly address the size of tumor lesion. Indeed, backscattered signals depend on the number of fluorescent cells, the intracellular level of fluorescent protein expression and the thickness of embryo tissues. Additional imaging modality is required to fulfill this need.

We are fully aware that the model proposed here does not fully recapitulate human disease. Indeed, the metastatic process may be stimulated by the chick embryonic environment and the very short time leading to metastasis formation most likely influences the final features of tumors. However, our results highlight the potential utility of the chick embryo CAM model as an in vivo tool to assess tumor sensitivity to therapeutic compounds. Highly sensitive 3D fluorescence and CT imaging allows the localization of metastasis in the chick embryo but also gives possibilities to quantify the metastatic foci signals in comparison to negative controls or treated chick embryos. Thus the CAM system emerges as a complementary assay for drug testing.

Recently, optimization of 3D primary cultures has provided biologically relevant information on tumor growth and response to various stimuli^32^. However, the complex interactions within the organism are hardly mimicked in 3D co-culture techniques^2^ and complementary preclinical tools are needed. Up until now, many successful drug tests were performed on primary nodules of the CAM and confirm utilization of this model as a reliable preclinical model for testing novel therapeutics^17,26,33^. The effect of nanoparticle-based anticancer drugs on ovarian cancer cells in the CAM model has also been reported^18^. Here we focus on targeting metastatic capacities of tumor cells in the chick embryo. First, we demonstrate the effects of common chemotherapeutics such as docetaxel and cisplatin on prostate and lung cancer metastatic growths. Then we mimicked the response of *ALK*-positive NSCLC to tyrosine kinase inhibitor crizotinib. The pharmacological growth inhibition of pancreatic ductal adenocarcinoma by crizotinib was previously reported by measuring luciferase activity in primary nodules on the CAM^11^. The possibility to use a CAM model to test targeted therapies was confirmed quantitatively using two others molecules, namely gefitinib (EGFR inhibitor) and enzalutamide (androgen receptor inhibitor) to treat eggs implanted with H1650 and LNCaP cells respectively. This suggests that, with the combination of fluorescence and 3D CT whole-embryo imaging, the chick embryo assay may serve as a rapid and efficient method to perform a first screening of potential therapeutic molecules.

One of the seldom recapitulated events in metastatic models is tumor tissue tropism^34^. Indeed, specific bone tropism of advanced PCa hardly occurs in spontaneous PCa models. Up until now, the most representative models were obtained after intracardiac or intraosseous injection of tumor cells in immunodeficient mice^35^ or, as recently reported, in bioengineered mouse models with humanized bones^36^. Recently CAM xenografts from PCa were successfully established^37^. Previous studies on PCa using the CAM were based on visualization of the primary tumor and intravasation process^9^. The metastatic foci of PC3 PCa cells were found in the brain of chick embryos^17^ and bone evaluation was not mentioned. In this study, the most aggressive IGR-CaP1 and GR-CDX P1 PCa cell lines preferentially formed metastases in the developing bones of the chick embryo, which suggests the relevance of the CAM in modeling PCa metastatic progression and its tropism.

### Conclusion

The chick embryo model is a powerful tool to improve understanding of basic principles of metastatic colonization with new possibilities for improving precision medicine approaches for metastatic cancer patients. This report shows that the CAM model, formerly used in embryogenesis and angiogenesis studies, provides a platform for functional characterization of metastatic tumors and therapeutic compound screening approaches when combined with advanced CT and spectral imaging. Indeed, evaluation of cancer spread beyond the primary lesion using sensitive imaging technologies continues to improve today. In addition to preclinical studies, future work should focus on using the CAM as a platform to graft different primary cell and tissue types, which has already been started^6,24,37^.

## Methods

### Cell cultures

All cell lines, except IGR-CaP1-GFP, LNCaP-GFP and GR-CDX P1-GFP, were obtained from ATCC collection and maintained in RPMI medium supplemented with 1% penicillin/streptomycin and 10% fetal bovine serum at 37 °C in 5% CO_2_. LNCaP-GFP and IGR-CaP1-GFP were a gift from A Chauchereau^19,20^ and GR-CDX P1-GFP cell line was established and characterized in the laboratory^23^.

### The chick embryo metastasis model

Fertilized chicken eggs were purchased from a certified hatchery and incubated for 3 days at 37 °C with 60% humidity. At ID 3, eggs were cleaned and a window of an approximate 2 cm-diameter was drilled into the eggshell to lower the CAM by creating an air pocket between the separated shell membrane and the CAM. This window allows to manipulate and implant the cells. Before closing it, 100 µL of penicillin (10^4^ U/mL) and streptomycin (10 mg/mL) were added into the CAM. The whole procedure was performed under aseptic conditions. The window was then covered with parafilm and the egg was placed back in the incubator. At ID 10, 10^3^ (unless stated differently) tumor cells per egg diluted in 20 µL of medium were mixed with 20 µL of matrigel. The mix was incubated as a drop for 30 min at 37 °C. The CAM was lacerated gently using a sterile cotton swab to facilitate engraftment, and the semisolid mix of matrigel and cells was subsequently implanted at the place of laceration into the CAM. Again the window was covered with parafilm and the egg was placed back in the incubator. Tumor growth and embryo viability were examined daily until the day of imaging analysis i.e. ID 17. At ID 17 (day 7 post-implantation) the complete analysis of tumor and embryo was performed.

### Fluorescent Macroscopy imaging

Fluorescent tumor nodules were visualized in vivo using the AZ100 fluorescent macroscope (Nikon, Tokyo, JP) equipped with the NIS software. GFP- and mCherry-expressing cells in the primary tumor nodules were imaged on the CAM using Ex 482/35 nm, DM506, Em 536/40 nm and ex543/22 nm, DM562, Em593/40 filter sets respectively.

### Whole 3D-chick embryo Fluorescence imaging and quantification

Chick embryos were scanned post mortem for fluorescence detection. Fluorescence and CT scans were performed simultaneously using the IVIS Spectrum Imager (PerkinElmer, MA, USA). The system is equipped with 10 narrow band excitation filters (30 nm bandwidth) and 18 narrow band emission filters (20 nm bandwidth) and gives possibility to read fluorescence in spanning wavelengths: 430–840 nm. 2D fluorescent images were acquired in epi-illumination using the adequate filters for GFP or mCherry fluorophores and displayed as an overlay of a black/white photograph and fluorescent signals. In 3D, the chick embryo was first scanned in CT before being examined in trans-illumination through 15 spots of excitation (with GFP or mCherry excitation filters) on average and covering the entire chick embryo. For 2D and 3D images, fluorescent signals were acquired with a 12-cm field of view, a binning (resolution) factor of 8, and a 2/*f* stop-and-open filter. The acquisition time was automatically computed by the software in order to obtain a minimum of 600 counts/pixel. In post-treatment using the Living Image software, tumoral fluorescent foci were reconstructed on the CT images. Regions of interest (ROI) were then defined manually on images (using an adapted to embryo size area in each case), and signal intensities were calculated using the Living Image software (PerkinElmer, MA, USA) and expressed as radiant efficiency or absorbance for 2D or 3D respectively.

### Histochemistry

After imaging, the CAM tumor nodules were collected and fixed in 4% PFA (Electron Microscopy Sciences, Hatfield, PA) and embedded in paraffin after tissue processing. Serial 7-µm paraffin sections were processed and routinely stained with hematoxylin-eosin-safranin (HES). Immunohistochemistry staining was performed with antibodies mouse monoclonal anti-human Ki-67 (MIB 1; Dako M724001) and anti-human Vimentin (Roche, 790-2917).

### Isolation of genomic DNA from cells and tumor samples

Nodules and metastases from H1299 and H1975 cells were collected at ID 17. DNA from control cell lines, tumor nodules from the CAM and metastasis from chick embryo organs were isolated according to manufacturer protocols using DNeasy Blood and tissue Kit (Qiagen).

### Targeted next-generation sequencing

The extracted DNA was subjected to sequencing on selected amplicons for representative genes; the detailed process was described previously^38^. Two targeted panels were used: the Ampli1 Cancer Hotspot Panel Custom Beta adapted from Ion Ampliseq CHP v2 by Menarini Silicon Biosystems covering 2,265 COSMIC hotspots regions across 315 amplicons of 48 cancer-related genes and an in-house panel targeting the tyrosine kinase domain in *ALK* and *EGFR* genes. Sequencing was performed using appropriate Ion chips for the IonPersonal Genome Machine or the Ion S5 System.

### RNA extraction and qRT-PCR analysis

RNA was extracted from 10^6^ IGR-CaP1 cells for positive control and collected at ID 17 from chick embryo bones in which in the 3D scan imaging the fluorescent signal was overlapping with the CT image of bone. Bones were purified from muscle and connected tissue, then bone marrow was flushed and empty bone was crushed and put together with the bone marrow. RNA extraction was performed according to manufacturer protocol using RNAsy MicroKit (Qiagen) and reverse-transcribed using the Maxima First Strand cDNA Synthesis Kit (Thermo Scientific, K1622). qRT-PCR was performed using TaqMan Universal Master Mix (Applied Biosystems, Roche) and a CFX96 Touch Real-Time PCR Detection System (Bio-Rad). The TaqMan primers were obtained from Applied Biosystems (TNFRSF11B/OPG, Hs00900358_ml; SPP1/osteopontin, Hs00959010_ml; Vimentin, Hs00185584_ml) and were used as previously described^19^.

### Stable cell lines expressing mCherry

The stable tumor cell lines expressing mCherry were established after infection with mCherry-expressing retroviral vectors (plasmid: IRES-mCherry #80139, Addgene Teddington, UK). Production and titration of retroviral particles were performed as described ^39^. The infection was performed on retronectin-coated plates (TaKaRa Bio, CA, USA) and efficiency was assayed by testing mCherry expression using flow cytometry. When efficiency was below 98%, cell sorting was performed.

### PKH26 staining

PKH26 linker was purchased from Sigma (St.Louis, MO) and staining was performed according to manufacturer protocol. Briefly, 10^6^ cells were washed with PBS and resuspended in 500 µL of diluent buffer. 2 × Dye solution (500 nM) was prepared in 500 µL of diluent buffer. Two solutions were mixed and incubated 4 min at room temperature. Reaction was stopped by adding 1 mL of serum. Cells were subsequently washed and prepared for implantation. 2 × 10^5^ stained cells were incubated in vitro in standard culture conditions in order to verify the fluorescence signal daily throughout the experiment (7 days).

### Treatments

For dose determination, the viability of chick embryos and morphology-altering effects were systematically investigated, as reported elsewhere (11, 18, 26, 31). Cisplatin was tested at the doses of 50 µg/kg, 30 µg/kg and 10 µg/kg. Doses of 50 and 30 µg/kg were toxic for chick embryos (100% of embryos died at ID 15). All embryos were alive and no influence on their development was observed at the dose of 10 µg/kg which was selected for drug-assays. Docetaxel was tested at doses of 20, 10 and 2 µg/kg. Doses of 20 and 10 µg/kg were toxic for chick embryos and the 2 µg/kg dose was selected. Gefitinib and crizotinib were tested with doses of 20, 10 and 2 mg/kg. Important toxicity was observed with gefitinib doses of 20 mg/kg and 10 mg/kg (50% and 40% of embryos died respectively), and crizotinib dose of 20 mg/kg (60%). An important teratogenic effect was observed in 50% of eggs at crizotinib dose of 10 mg/kg. Dose of 2 mg/kg was selected for both gefitinib and crizotinib. Enzalutamide was toxic for chick embryos at 1 mg/kg (80%). The 400 µg/kg dose did not produce side effects and was selected. Tested eggs were monitored daily throughout the experiment. Chick embryo mobility as well as increasing vascularization of the CAM (the two indicators of chick embryo viability) were evaluated and compared to non-treated eggs.

Tumors originating from LNCaP, H1299, A549 cell lines were treated 24 h after cell implantation with vehicle, 2 µg/kg docetaxel, or 10 µg/kg cisplatin respectively. Tumors originating from H2228, H1650, DU145 and LNCaP were treated daily from ID 11 up to ID 16 with vehicle, 2 mg/kg crizotinib, 2 mg/kg gefitinib, or 400 µg/kg enzalutamide. The drug was administered topically on the CAM, near the tumor nodule and the final doses were calculated based on the weight of the chicken egg at ID 10.

### Statistical analysis

Data are shown as means and standard error of the mean (SEM), and P values are reported as follows: *P < 0.05, **P < 0.01, and ***P < 0.001. Statistical analysis was performed using the GraphPad Prism software, version 6.0 (GraphPad Software, San Diego, CA). Data were analyzed using nonparametric Mann–Whitney test.

## Supplementary information

Supplementary file1

Supplementary file2

Supplementary file3

Supplementary file4
