# Supplementary material for: Exploitation of the chick embryo chorioallantoic membrane (CAM) as a platform for anti-metastatic drug testing
Source: Sci Rep. 2020 Oct 9;10:16876. doi: 10.1038/s41598-020-73632-w (PMC7547099; doi:10.1038/s41598-020-73632-w)
Supplement: Supplementary file 1 — Supplementary file1 [file 41598_2020_73632_MOESM1_ESM.pdf]

Exploitation of the chick embryo chorioallantoic membrane (CAM) as a platform for anti-metastatic drug testing

Pawlikowska P, Tayoun T, Oulhen M, Faugeroux V, Rouffiac V, Aberlenc A, Pommier AL, Honore A, Marty V, Bawa O, Lacroix L, Scoazec JY, Chauchereau A, Laplace-Builhe C, Farace F

Supplementary Material

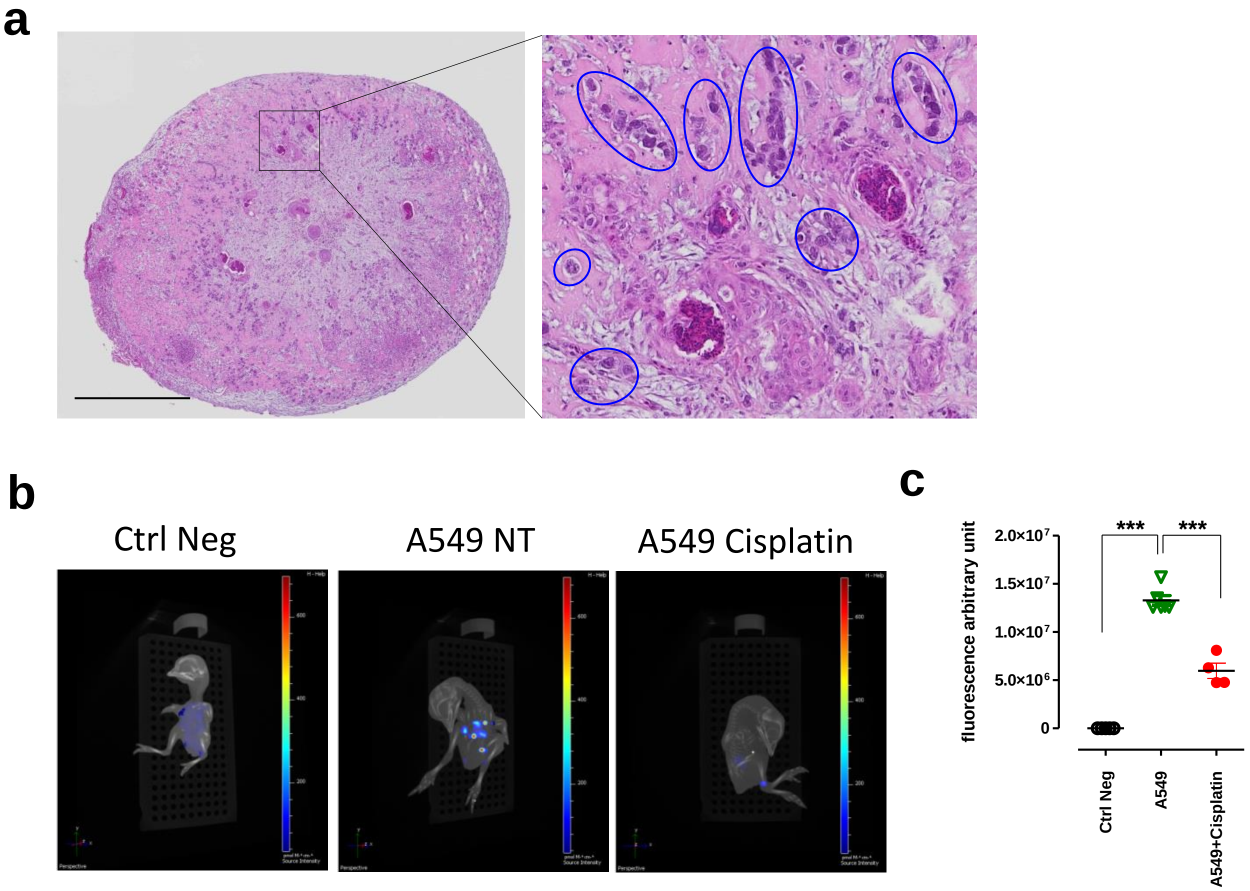

Supplementary Figure 1.

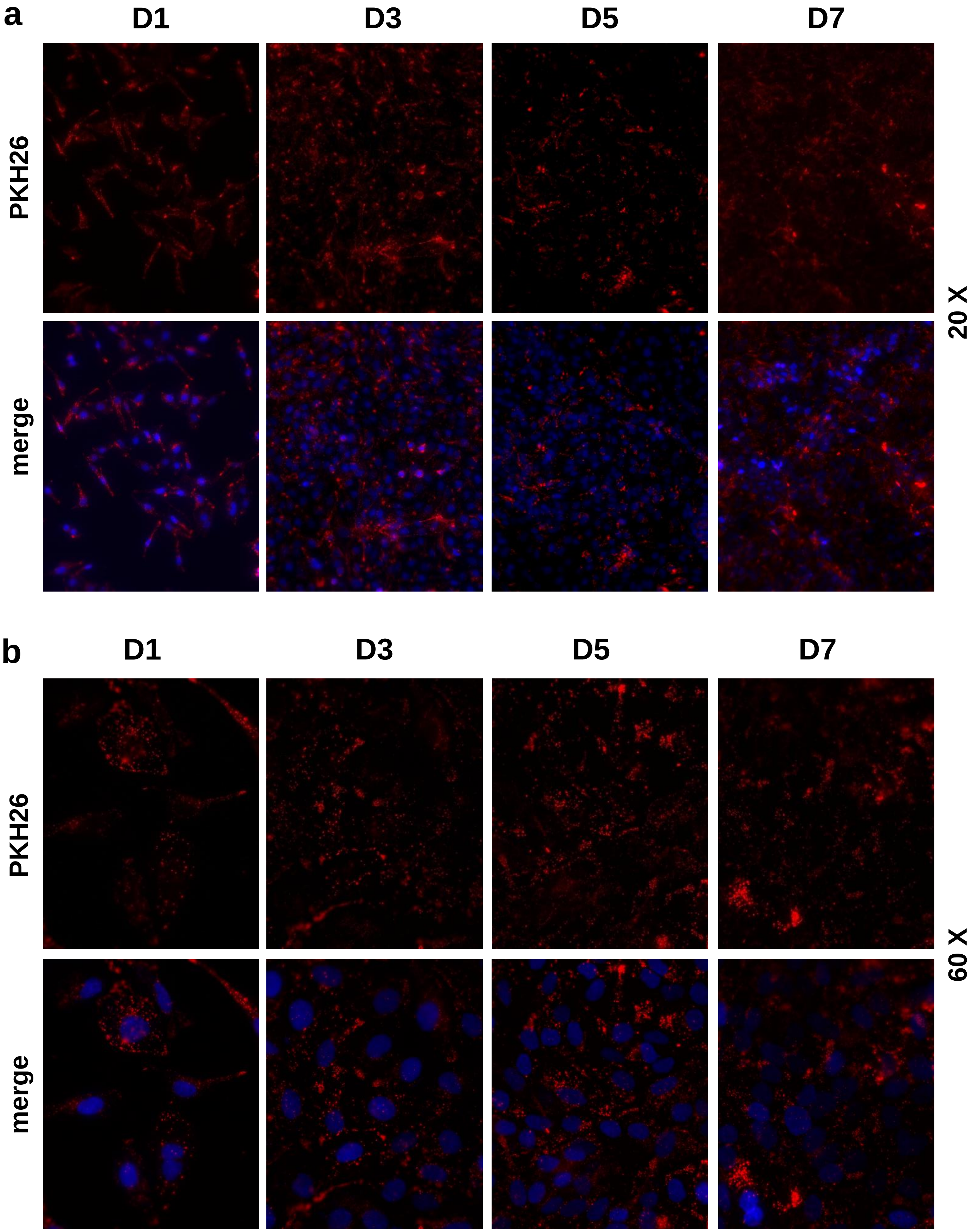

Supplementary Figure 2.

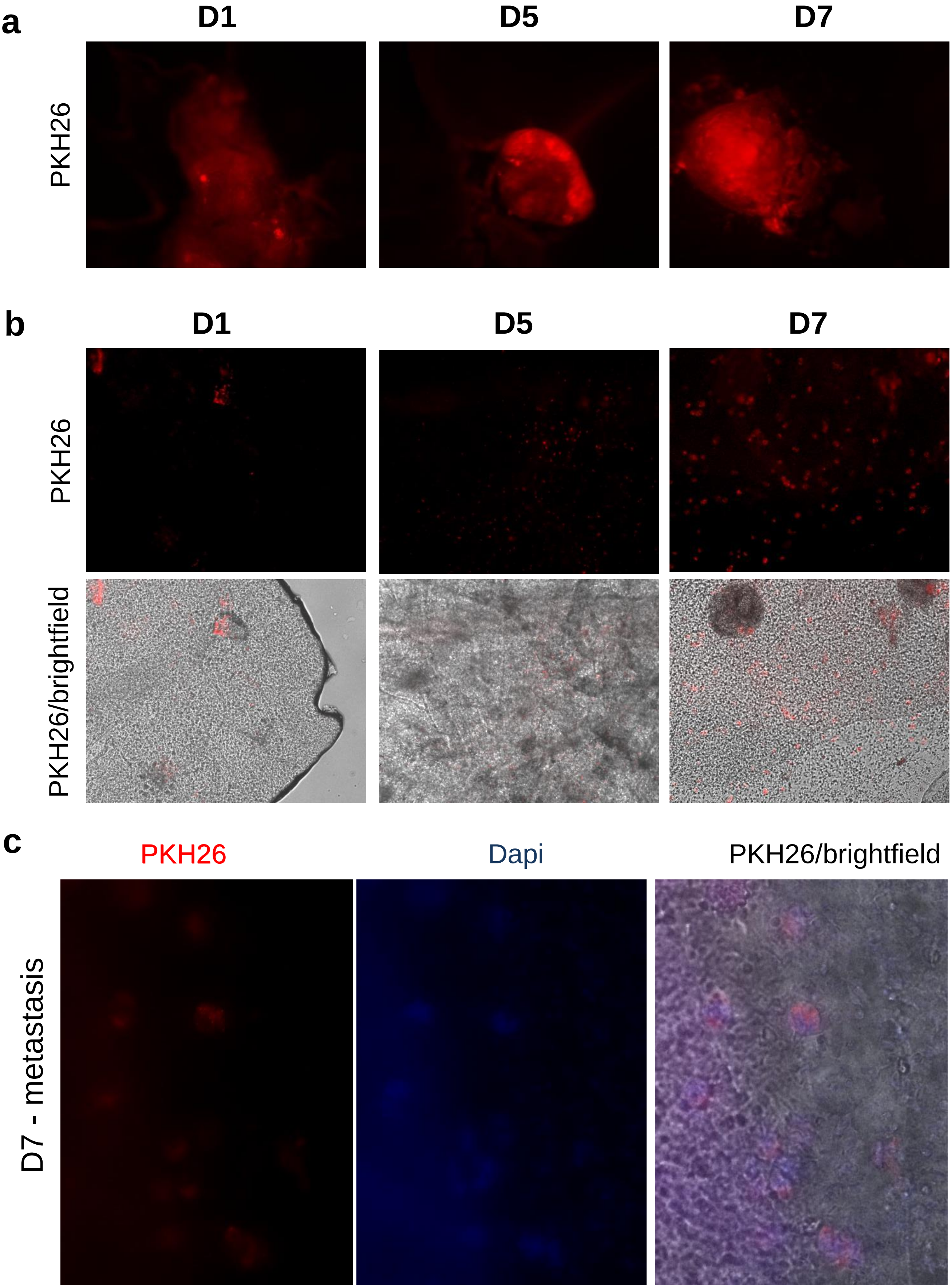

Supplementary Figure 3.

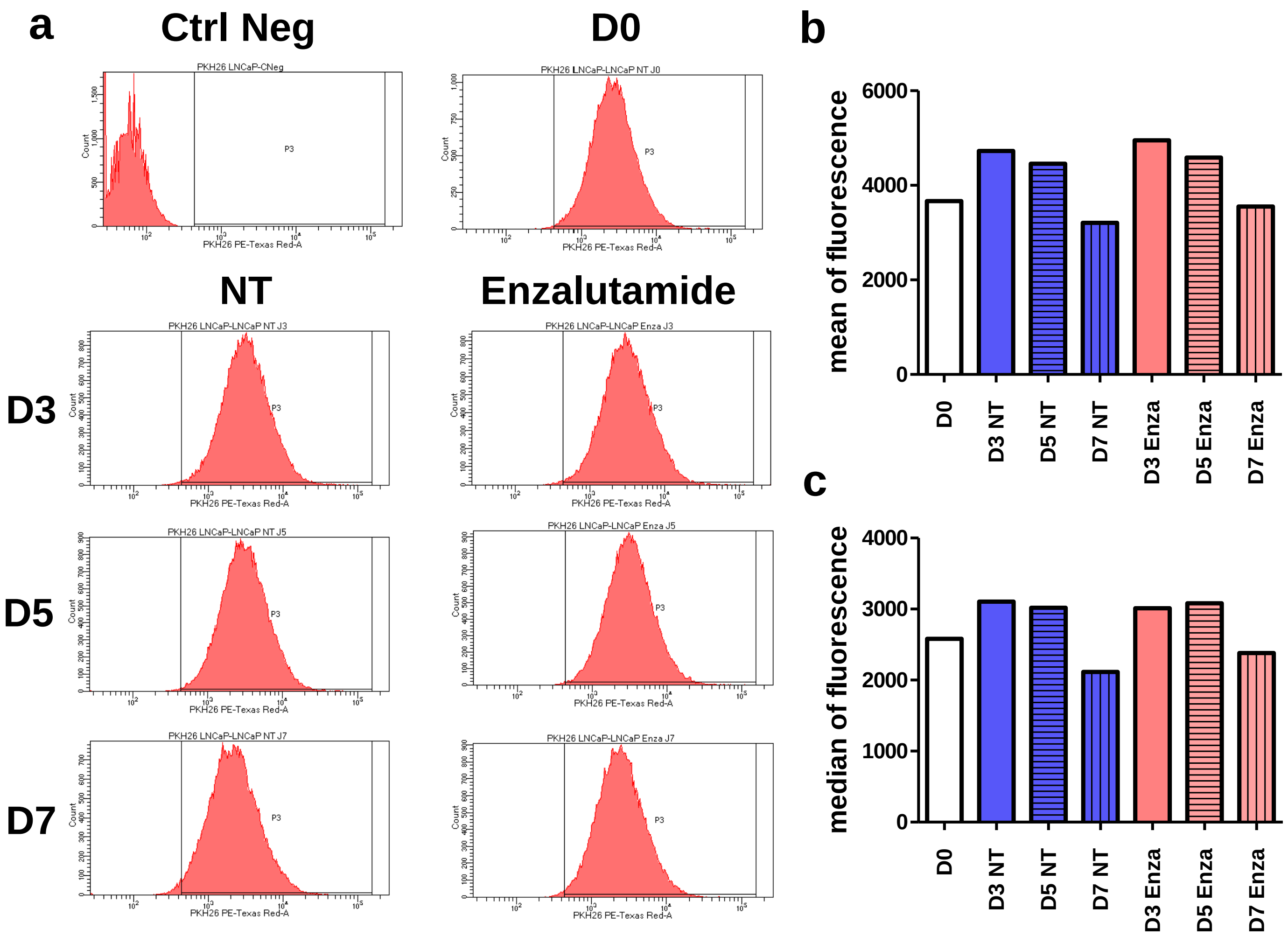

Supplementary Figure 4.

Supplementary Figures

Fig. 1. a. Tumor nodules of H1299-GFP, FFPE sections stained with HES. Areas with tumor cells were framed. b. Representative images of fluorescent metastatic foci formed by A549 mCherry cells with or without cisplatin treatment. c. Quantitative analysis of mean fluorescence from 3D analysis of chick embryos after implantation of A549 mCherry cells. Experiment was performed twice and the representative one is shown. Each point represents a single embryo.

Fig. 2. Representative images showing the pattern and intensity of PKH26 staining during 7 days of *in vitro* culture of LNCaP cell line. Magnification 20x a, and 60x b. Images were captured using NIS Elements software Version 4.0; AR Ver4.00.05 for 64bit edition (Nikon Instruments Inc., https://www.microscope.healthcare.nikon.com/products/software/nis-elements).

Fig. 3. Representative images showing a. tumor nodules obtained from LNCaP stained with PKH26 and collected at different time-points; examined *in vivo* by a fluorescence macroscope. Images were captured using NIS Elements software Version 3.2; BR Ver for 64bit edition (Nikon Instruments Inc., https://www.microscope.healthcare.nikon.com/products/software/nis-elements). b. PKH26 fluorescent cells presented in tumor nodules using fluorescent microscope (20x) with the corresponding bright field images. c. Example of metastatic foci in esophagus inner membrane of chick embryo 7 days after implantation of LNCaP cells stained with PKH26. Images obtained using fluorescent microscope (20x) with the corresponding bright field images. Images were captured using NIS Elements software Version 4.0; AR Ver4.00.05 for 64bit edition (Nikon Instruments Inc., https://www.microscope.healthcare.nikon.com/products/software/nis-elements).

Fig. 4. *In vitro* analysis of LNCaP cells stained at D0 with PKH26 and treated or not with enzalutamide (500 nM) during 7 days. PKH26 fluorescence intensity was measured by FACS. a. FACS histograms showing PKH26 fluorescence at different days of the experiment and negative control presenting non-stained cells. b. Graph representing mean of PKH26 fluorescence at each day of experiment. c. Graph representing median of PKH26 fluorescence at each day of experiment.

Cell line	Tumor type	alteration	origin
NCI-H1299	Large cell carcinoma	NRAS Q61K	ATCC
NCI-H1975	Non-small cell lung carcinoma	EGFR T790M<br>EGFR L858R<br>TP53 R273H<br>CDKN2A E69*	ATCC
A549	Non-small cell lung carcinoma	KRAS G12S	ATCC
NCI-H2228	Non-small cell lung carcinoma	EML4-ALK fusion	ATCC
NCI-H1650	Non-small cell lung carcinoma	EGFR delE746-A750	ATCC
LNCaP	Prostate carcinoma Androgen receptor positive	PTEN loss	ATCC
DU145	Prostate carcinoma Androgen-independent	8q gain	ATCC
IGR-CaP1	Prostate carcinoma	TP53 Y126C	[19]Al Nakouzi *et al.* 2012
GR-CDX P1	Prostate carcinoma	TP53, RB1, PTEN loss	[23]Faugeroux *et al.* 2020

Supplementary Table 1. List of used cell lines with their short characteristics.

Fluorescent protein/dye	Excitation (nm)	Emission (nm)	Advantages in context of CAM model	Disadvantages in context of CAM model
GFP	465	520	Very stable and non-toxic	Sensitivity of fluorescent imaging is limited by strong and heterogeneous auto-fluorescence signal of the chick embryo
mCherry	570	620	Compatible with deep imaging,<br>low auto-fluorescent background,<br>resistant to photo-bleaching	Possibly less stable than GFP
PKH26	535	580	Compatible with deep, imaging,<br>low auto-fluorescent background,<br>useful for *in vivo* long-lasting studies (here 7 days), compatible with 3D IVIS Spectrum Imaging	Toxicity, required dose adaptation to cell/tissue types,<br>Localized in lipid regions of the intracellular membranes

Supplementary Table 2. Advantages and disadvantages of used fluorescent markers in the contexts of the CAM model.
